# Direct observation of electronic-liquid-crystal phase transitions and their microscopic origin in La_1/3_Ca_2/3_MnO_3_

**DOI:** 10.1038/srep37624

**Published:** 2016-11-22

**Authors:** J. Tao, K. Sun, W.-G. Yin, L. Wu, H. Xin, J. G. Wen, W. Luo, S. J. Pennycook, J. M. Tranquada, Y. Zhu

**Affiliations:** 1 Condensed Matter Physics & Materials Science Department, Brookhaven National Laboratory, Upton, NY 11973, USA; 2 Department of Physics, University of Michigan, Ann Arbor, MI 48109, USA; 3 Center for Functional Nanomaterials, Brookhaven National Laboratory, Upton, NY 11973, USA; 4 Center for Nanoscale Materials, Argonne National Laboratory, 9700 S. Cass Avenue, Argonne, IL 60439, USA; 5 Department of Physics & Astronomy, Shanghai JiaoTong University, Shanghai, China; 6 Department of Materials Science and Engineering, National University of Singapore, 119077 Singapore

## Abstract

The ground-state electronic order in doped manganites is frequently associated with a lattice modulation, contributing to their many interesting properties. However, measuring the thermal evolution of the lattice superstructure with reciprocal-space probes alone can lead to ambiguous results with competing interpretations. Here we provide direct observations of the evolution of the superstructure in La_1/3_Ca_2/3_MnO_3_ in real space, as well as reciprocal space, using transmission electron microscopic (TEM) techniques. We show that the transitions are the consequence of a proliferation of dislocations plus electronic phase separation. The resulting states are well described by the symmetries associated with electronic-liquid-crystal (ELC) phases. Moreover, our results resolve the long-standing controversy over the origin of the incommensurate superstructure and suggest a new structural model that is consistent with recent theoretical calculations.

The analysis of a phase transition is relatively straightforward when there is a well-defined order parameter. In the absence of long-range order, interpreting the nature of a phase transition can be challenging, as multiple possible causes of disorder can lead to considerable ambiguity. For instance, the intriguing properties of doped manganites, such as colossal magnetoresistance (CMR)^1^ and giant magnetocaloric effects^2^, are associated with a rich variety of competing electronic phases. Taking La_1−*x*_Ca_*x*_MnO_3_ (0.5 ≤ *x* ≤ 0.8) as an example, charge order was widely observed to be associated with a long-range (LR) superstructural modulation at low temperatures^3^,^4^,^5^,^6^,^7^,^8^,^9^,^10^,^11^,^12^,^13^,^14^. This electronic superstructure arises from the competition of spin, charge, orbital and lattice degrees of freedom and is of great interest in understanding many properties in doped manganites. However, there are controversies over the interpretation of the various characterizations of the lattice modulation and its thermal evolution as the charge order dissipates. One controversy is associated with the precise determination of the phase transition temperature, which depends on the quantity that is used to characterize the transition. By using diffraction techniques, the intensity, width, and position of the superlattice reflections (SLRs) of the lattice modulation exhibit distinct behaviors with temperature. In particular, the intensity and width indicate a continuous transition from LR to short-range (SR) order upon warming; without a sharp transition, the determination of the transition temperature is somewhat arbitrary. Meanwhile, measurements of the SLR peak position reveal a commensurate to incommensurate (C-IC) phase transition upon warming with a clear transition temperature. The relationship between those characterizations of the phase transition, i.e., the LR-SR and the C-IC transitions, remains uncertain^6^.

These apparently conflicting behaviors are not easily reconciled with common models of a phase transition in a homogeneous system, and they have contributed to an intensive debate^1^,^4^,^6^,^7^,^8^,^9^,^10^,^11^,^12^,^13^,^14^,^15^,^16^. In the literature, the structural modulation of doped manganites is often considered to involve charge ordering (CO) and orbital ordering (OO), i.e., Mn ions have two valence states (Mn^3+^ and Mn^4+^) and Mn^3+^ has one localized *e*_*g*_ electron^3^,^4^,^5^,^6^,^9^,^10^,^11^,^12^; in other approaches, it is argued that there is no charge difference between the two types of Mn ions, but instead the modulation is a consequence of charge-density-wave or simply orbital ordering^13^,^14^,^15^,^16^,^17^. In the latter camp, a central argument is that the CO/OO model can explain commensurate superstructure but is not compatible with incommensurate superstructures^13^. Alternatively, electronic defects such as discommensurations^6^ or solitons^7^ in the CO/OO model were proposed to be responsible to the incommensuration. However, the evolution of those defects during the transition still lacks direct observation. Thus, the role of defects in the superstructure and the phase transition is not clear. Real-space observations of the evolution of the superstructure through the LR-SR and C-IC phase transitions are urgently needed to understand the underlying physics in doped manganites.

New options for interpreting the phase transitions are provided by the proposal of electronic-liquid-crystal (ELC) phases^18^,^19^,^20^ which has been developed to describe electronic textures in correlated materials. For example, the concepts of electronic smectic and nematic phases have been applied to cuprate^20^,^21^,^22^,^23^,^24^ and Fe-based^25^,^26^ superconductors, bilayer ruthenates^27^, a two-dimensional electron gas under high magnetic field^28^, fractional quantum Hall systems^29^, and a “nematic-like” phase found in a doped manganite^30^; even nematic insulators have been considered^31^,^32^. ELC phases were first introduced to describe electronic fluids in high temperature superconductors and focused on the electronic structures, especially those arising from the spin order^18^,^20^. The latter idea was generalized as a method to classify different electronic states of matter based on the spatial symmetry breaking patterns. During the development of the ELC theory, it has been realized that the symmetry breaking of the crystal lattice and the electronic correlations may be difficult to separate^19^ and that other degrees of freedom, such as orbitals, may play a role. One area that has not yet received experimental attention is the smectic-nematic transition. Anticipating that the transition takes place though a proliferation of dislocations, it is essential to have real-space information to characterize it. Furthermore, it is imperative to work with a 3D material, so that the smectic order is not destroyed by the random-ion disorder commonly associated with electronic doping.

In this article, we use advanced transmission electron microscopy (TEM) techniques to detect two transitions of the superstructure in La_1/3_Ca_2/3_MnO_3_ (a 3D material compound), in place of the one ambiguously-determined transition previously reported^4^,^5^,^6^,^9^. These transitions, identified as electronic smectic-nematic and electronic nematic-isotropic, are precisely characterized and analyzed. In particular, with help from recently developed ELC theory, the observed smectic-nematic transition provides the physical origin of both the LR-SR transition and the C-IC transition. These findings not only shed light onto the nature of the superstructures in La_1−*x*_Ca_*x*_MnO_3_, but also provide a novel example of ELC phenomenology.

## Results and Discussion

**Figure 1:**
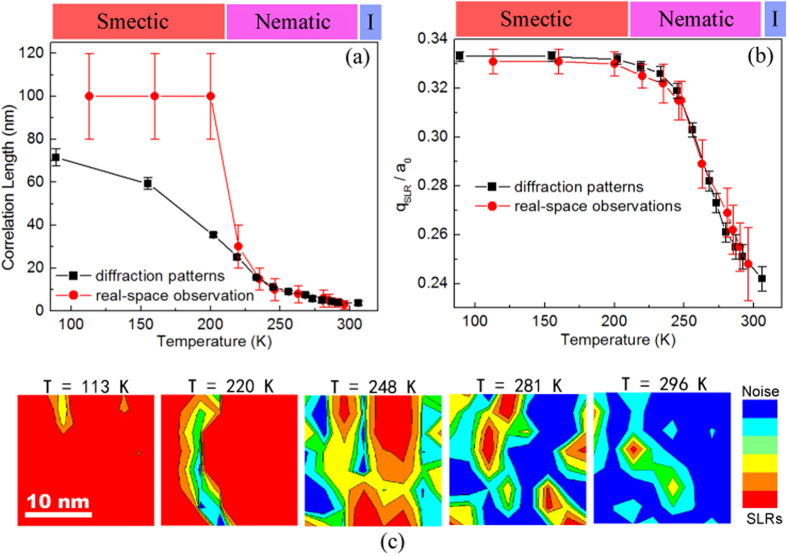
The observations of electronic smectic-nematic and nematic-isotropic (I) transitions in La_1/3_Ca_2/3_MnO_3_. (**a**) The correlation length as a function of temperature. The black squares were measured from the full width at half maximum of the SLRs in the volume-averaged ED patterns at [010] zone. The red dots were estimated directly from SEND maps in (c). (**b**) The wavenumber of the SLRs measured from ED patterns (black squares) and END patterns (red dots) as a function of temperature. (**c**) SEND maps the SLRs’ intensity obtained from END patterns scanned over in a single-crystal domain in the La_1/3_Ca_2/3_MnO_3_ material (see ref. 32 for the principle of the technique). Warm colors represent the superstructure order, while cold colors correspond to superstructure disorder. The maps were not registered to each other in position.

La_1/3_Ca_2/3_MnO_3_ is known to have a unidirectional superstructure aligned along the *a*-axis at low temperatures^3^,^4^,^5^. The fundamental lattice at room-temperature and the superstructure at low temperatures are both orthorhombic with the space group of *Pnma*^4^,^5^. From the ELC perspective, the superstructure, with a periodicity 3 lattice spacings, breaks the translational symmetry along one direction with respect to the fundamental lattice. With distinct structures along the *a* and *c* axes compared to the fundamental lattice, the superstructure breaks the point group rotational symmetry, as well (see supplementary material for details on the classification of ELC phases). Therefore, this electronic phase with LR superstructure can be classified as an electronic smectic. The superstructure can be probed by the SLRs either in electron diffraction (ED) patterns obtained from a large volume of the material or in electron nanodiffraction (END) patterns using an electron beam smaller than 2 nm in diameter (Fig. S1). The correlation length, measured from the width of the SLRs in the ED patterns (black symbols in Fig. 1a), decreases from ~70 nm at *T* = 98 K to ~4 nm at *T* = 306 K, suggesting that the superstructure loses its LR coherence upon warming but without a sharp signature of a transition. On the other hand, the SLRs shift from commensurate (*q* = 0.33 in this case) to incommensurate (C-IC transition; see supplementary material for more discussions in the C-IC transition) at *T*_*1*_ = 210 ± 10 K (Fig. 1b) upon warming, measured from both the ED and the END patterns (see supplementary material for the measurements using electron diffraction and synchrotron x-ray scattering).

To identify the temperature at which the superstructure transforms from LR-SR, we utilized scanning electron nanodiffraction (SEND) imaging^33^ to map the intensity of the SLRs in the END patterns in real space; the spatial distribution of the superstructure order (red) is shown in Fig. 1c. The superstructure order is LR at low *T*~113 K, shown by the homogeneously distributed red color in the scanned area. The intensity fluctuation in minor regions at the top of the SEND map at 113 K is comparable to the measurement uncertainty. The superstructure map starts to break into separated areas above *T*~210 K, and the superstructure regions continue to shrink on further warming. To obtain a measure of this change, the correlation length of the ordered areas in the SEND maps is indicated by the red dots in Fig. 1a, revealing a sharp transition at *T*_*1*_ = 210 ± 10 K, beyond which the disordered regions start to percolate and the ordered regions become isolated. We note firstly that our real-space characterization yields a LR-SR transition temperature that is the same as that for the C-IC transition, in contrast to the results from the spatially-averaged diffraction measurements in ref. 6. Secondly, because there is no LR superstructure at *T* > *T*_*1*_, the translational symmetry of the electronic structure for the bulk is effectively the same as the fundamental lattice and only the rotational symmetry of the electronic superstructure remains broken with respect to the fundamental lattice. Namely the electronic phase as a whole in the bulk has a LR nematic order. At *T* > *T*_*2*_~310 K, the SLRs have undetectable intensities and the electronic structure transforms into an isotropic phase, with the same translational and rotational symmetry as the fundamental lattice. Therefore, our observations identify an electronic smectic-nematic transition at *T*_*1*_~210 K and an electronic nematic-isotropic transition at *T*_*2*_~310 K in La_1/3_Ca_2/3_MnO_3_.

The coincidence of the electronic smectic-nematic transition (the same as the LR-SR transition by the definition) with the C-IC transition stimulated further exploration to understand the relationship. A recent work of Nie and coworkers^34^ indicates that, for a 3D system at finite temperature, a commensurate stripe phase is stable against weak disorder (likely to be charge disorders) and therefore is consistent with smectic order, but an incommensurate stripe phase is not; the resulting “vestigial order” is a nematic, consisting of SR incommensurate stripes and a disordered counterpart^34^. Our experimental observations present a concrete example of that theoretical proposal. More interestingly, an empirical rule *q* = 1 − *x*, where *q* is the wave number of the superstructure and *x* is the doping level, was found to describe the ground state of LCMO crystals at both commensurate or incommensurate doping levels^3^. Accordingly the effective doping level *x*_*eff*_, derived from *x*_*eff*_ = 1 − *q*, inside the ordered nano-regions is 0.75 ± 0.02 at 295 K, significantly larger than the nominal doping of 0.67. Note that the variation in *q* with temperature is seen consistently in both the real-space and spatially averaged measurements. Charge neutrality would require that the increase in doping of the ordered regions be compensated by a reduction in the disordered regions. We will return to the point later and present evidence for corresponding charge inhomogeneity.

**Figure 2:**
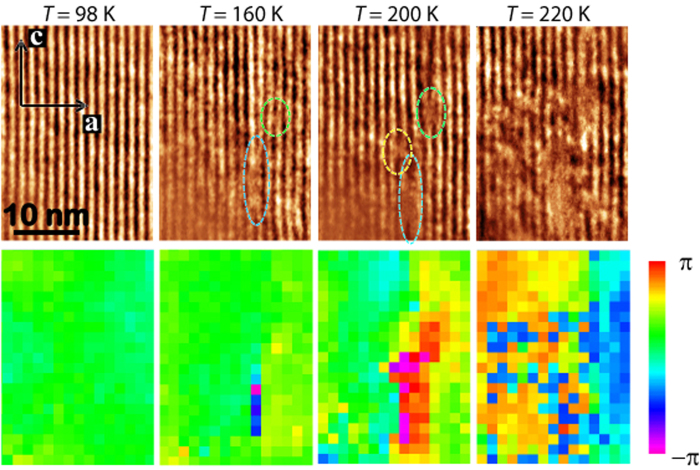
Top: A sequence of snapshots of dark-field TEM images recorded at different temperatures upon warming in La_1/3_Ca_2/3_MnO_3_. With the nearby crystal boundary as a marker (see Fig. S2), all the TEM images shown here were well registered in position. The formation of dislocation pairs was highlighted by dashed ellipsis. Bottom: Relative phase map extracted from the dark-field TEM images by performing fast-Fourier transform, selecting side band, reconstructing phase map using the Gatan DigitalMicrograph software with a scale bar for the reconstructed phase. Each phase map is corresponding to the dark-field image on the top.

To characterize the evolution of defects in the superstructure across the smectic-nematic transition, we performed dark-field imaging as shown in the top row of Fig. 2. At a temperature far below the transition, the superstructure order exhibits uniform stripe-like contrast in the *a-c* plane. On warming to 160 K, two pairs of dislocations can be seen (highlighted by blue and green dashed ellipses), with each pair appearing to break a stripe from the middle. At 200 K, just below the transition, an additional dislocation pair can be seen (yellow dashed ellipse) and the spatial separation of a pair has increased (green ellipse). When the temperature is above *T*_*1*_, the proliferation of defect destroys the periodic order, making it impossible to distinguish individual dislocations.

The coherence of the superstructure can be better visualized by mapping the phase function of the superstructure, as done in the bottom row of Fig. 2 (see more details in supplementary Fig. S3). Clearly, the appearance of discrete dislocations has little effect on the long-range phase order below the electronic smectic-nematic transition; it is responsible only for local phase discrepancies from the uniform background. Because the dislocations always appear to form in pairs, each pair can be treated as a local singularity that does not affect the LR coherence of the superstructure. Indeed, the proliferation of the dislocations at 220 K causes the percolation of the disordered region, corresponding to the transition to the nematic phase. These observations provide the first direct confirmation of the key role of dislocations, as proposed by the ELC theory more than a decade ago^18^.

**Figure 3:**
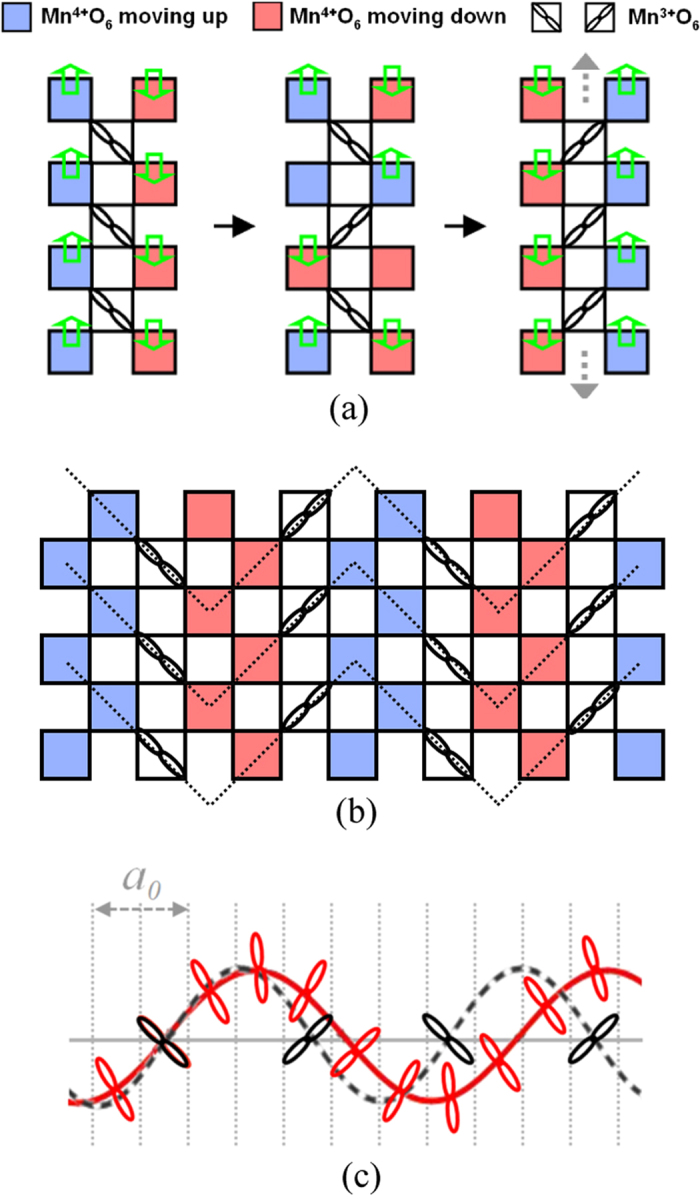
(**a**) A model proposes a possible formation mechanism of dislocations that it starts with 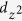 orbital disorder in a Mn^3+^ O_6_ octahedron and propagates along one stripe via elastic chain reaction. Mn^3+^ O_6_ and Mn^4+^ O_6_ only denote the octahedra with distinct orbital configurations, but do not reflect the charge valence of the Mn ions. (**b**) A classical model for the superstructure with orbital ordering. The dash lines are the CE-type chains for the magnetic order which forms at temperature ~150 K. (**c**) A schematic drawing demonstrates the models for the commensurate superstructure (black orbitals on the black dash curve) and pure incommensurate superstructure (red orbitals on the solid red curve) along the *a* axis. In the commensurate superstructure, the *e*_*g*_ orbitals have only two fixed orientations at the so-called Mn^3+^ O_4_ octahedra. In the incommensurate superstructure, the *e*_*g*_ orbital with certain occupied probability at each MnO_4_ octahedron rotates continuously. The sinusoidal curves indicate the amplitude of the atomic displacements of the Mn ions along the *a* axis.

As mentioned before, there has been a long and lively debate over the nature of the superstructure modulation in doped manganites with *x* ≥ 0.5^1^,^4^,^6^,^7^,^8^,^9^,^10^,^11^,^12^,^13^,^14^,^15^,^16^. For *x* = 0.67, in particular, neutron^4^ and electron^3^,^6^,^9^,^10^ diffraction studies provide strong evidence for a stripe-like order involving individual rows of Mn^3+^ separated by double rows of Mn^4+^ ions (corresponding to inserting “solitons” of Mn^4+^ into the a so-called CE-type state that occurs for *x* = 0.5)^7^. The extra electron on each Mn^3+^ site is associated with either a 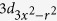 or 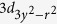 Wannier orbital (with substantial weight on neighboring O atoms)^11^,^12^, resulting in large Mn-O bond length splittings of 0.1 Å^4^. The modulation wave vector is oriented along the orthorhombic *a* axis, but much of the displacements are transverse to that direction. This ordered state is supported by theoretical calculations^14^,^35^; the superstructure modulation is illustrated in Fig. 3b. The controversy is largely associated with the thermally- or doping-induced C-IC transition^6^,^7^,^8^,^13^ and with disputed reports of sliding charge-density waves^15^,^16^. Those phenomena have led to proposals that the modulation might involve a relatively weak, uniform variation of charge^8^, as in a charge-density wave, or the development of discommensurations due to competing order parameters^6^,^8^.

Our observation that the commensurate-incommensurate transition occurs via dislocations is compatible with the model of stripe-like order in the commensurate phase. A model for the formation of the dislocation pairs in the superstructure is shown in Fig. 3a, with the defect-free superstructure demonstrated in Fig. 3b. We propose, as illustrated in the middle panel of Fig. 3a, that a thermal excitation can cause a defect in which the electron on one 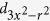 orbital hops to 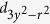 orbital by thermal excitation, along with the elastic distortions of four neighboring MnO_6_ octahedra. This configuration will certainly cost energy due to the elastic strain relative to the orbitally-ordered MnO_6_ octahedra above and below it; however, it should cost relatively little energy to extend this defect along the stripe direction, allowing the pair of dislocations to separate. The entropic free-energy gain from such configurations may compensate for the elastic-energy costs. We want to emphasize here that the dislocations observed in Fig. 2 are defects in the electronic superstructure and seem to be edge-type dislocations. As suggested by our model (Fig. 3a), dislocations in the superstructure do not necessarily indicate defects in the crystal lattice. Indeed, based on the thermal evolution of dislocations in the smectic phase shown in Fig. 2, the dislocations in the superstructure are most likely not related to defects of the average crystal structure. Our observations demonstrate that orbital order can play a dominant role in the LR commensurate superstructures.

In the nematic phase observed at temperatures above *T*_*1*_~210 K, dislocations are no longer relevant as the superstructure exists only in isolated areas. These isolated areas continuously shrink in size with warming, and the superstructure in these confined areas appears to be truly incommensurate, without the appearance of any dislocations^9^. This indicates that the origin of the incommensurability measured in the nematic phase cannot be defects in the superstructure, such as the previously proposed as discommensurations^6^, but must, instead, be intrinsic in nature. Charge-density-wave (CDW) order was once hypothesized to be the possible origin of the uniformly incommensurate superstructure in La_1−*x*_Ca_*x*_MnO_3_^13^,^15^. However, as we demonstrated above, the direct imaging of the superstructure here comes from the orbital contribution, and we propose a new model for the incommensurate superstructure, shown in Fig. 3c. The original model for the charge and orbital ordering structure with a commensurate three lattice-spacing period has the 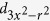 or 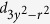 orbital located only at so-called Mn^3+^ O_3_ octahedra, shown in Fig. 3b. These orbitals can be considered as the combination of the 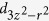 and 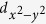 orbitals with the orbital mixing angle θ = 120° or −120° in the formula 
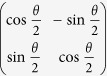
 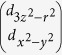. In the incommensurate superstructure model shown in Fig. 3c, the orbital mixing angle θ can vary continuously from one column of MnO_3_ octahedra to the next, so that the orientation of the *e*_*g*_ orbital rotates as one progresses along the *a* axis. The sinusoidal variation of the amplitude of the atomic displacements (transverse) along the *a* axis is only the simplest model that we could propose, and it can be refined with further experimental evidence and theoretical insight (see supplementary discussion section 4).

We emphasize that the proposed model here is very distinct from the conventional concept of the ordered orbitals, in which they must be aligned with the Jahn-Teller distortions in doped manganites. Theoretical calculations have already pointed out that the conventional understanding of the electronic structures by electron-lattice coupling alone, i.e., directly relating orbital ordering with Jahn-Teller distortion in a linear energy term, could be “misleading” or “insufficient to stabilize the orbital ordered state”^11^,^36^. Those calculations indicate that a better approach is to include electron-electron coupling and to take self-consistency into account to determine the electronic structure at a more realistic level^11^,^36^. As a result of self-consistency, it is possible to expect various angles between the driving Jahn-Teller distortion directions and the orientations of the consequently ordered orbitals. This is consistent with the model we have proposed here and is supported by our direct observations in real-space. A good analogy to such a mechanism may be the canted spin ordering in many antiferromagnetic systems, where the directions of the spins cannot be driven by the magnetic field alone, but also involve the spin-spin interactions, especially when the driving magnetic fields are not strong. The orbital degree of freedom, sometimes described in terms of a pseudo-spin, is demonstrated here to resemble actual spin ordering scenarios, such as a spin-density-wave (SDW).

**Figure 4:**
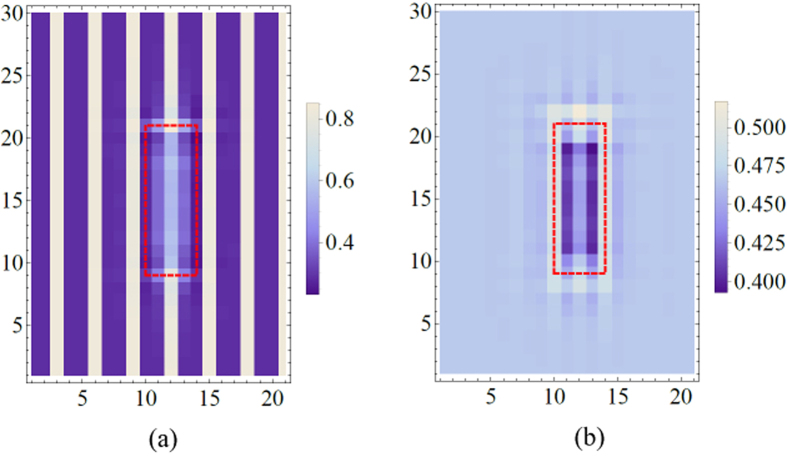
(**a**) Electron density for each unit cell and (**b**) average local densities for blocks with the size of 3 × 3 unit cells computed using the GL theory.

To further explore the mechanism of the smectic-nematic transition, a mean-field theory has been employed to test a charge-only version of the model in Fig. 3a. We first constructed a Ginzburg-Landau (GL) free energy which prefers unidirectional density-wave order along the *a*-axis (see supplemental material for details). In a region marked by the red box in Fig. 4a, the sign of the anisotropy in the free energy is reversed so that the perpendicular stripe direction is locally preferred. By numerically minimizing the GL free energy, the order within the red box is reduced, similar to the appearance of a pair of dislocations (Fig. 4a). The difference in order parameter between regions inside and outside of the red box should result in variations in local charge density. To visualize this effect, we compute the local charge density, yielding the results in Fig. 4b. Interestingly, we find that the average charge density within the red box is about 10% lower than the surrounding ordered area (for the parameters used in the calculation). When the disordered patches are small, the shift in the average charge density should be small, but when they proliferate, the increased charge density in the regions with the superstructure order should become noticeable, resulting in an increase in *x*_*eff*_, qualitatively consistent with the observed change in *q* (Fig. 1b).

**Figure 5:**
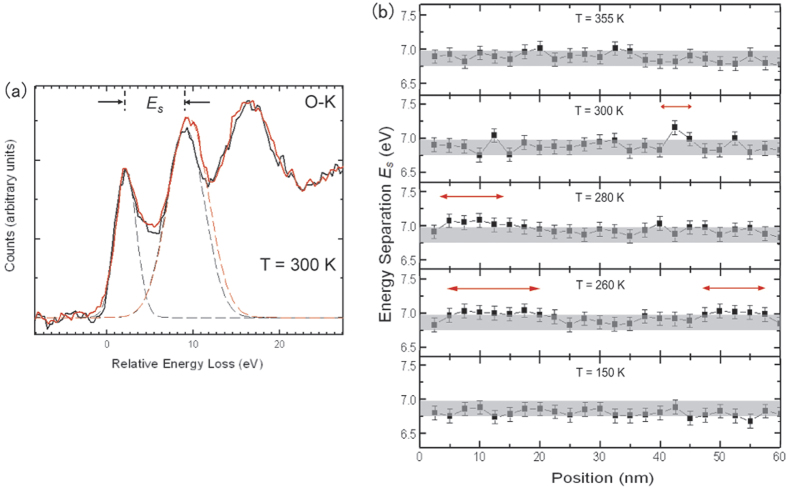
(**a**) Two typical EELS at O-K edge collected using an electron probe ~1 nm in diameter during scanning over La_1/3_Ca_2/3_MnO_3_. The pre-peak and main-peak energy separation *E*_*s*_ are evidently different for the two sets of spectra and corresponding to distinct *x*_*eff*_ values (using the fitting line in Fig. S5b). (**b**) One-dimensional real-space mapping of the *E*_*s*_ measured from EELS line scan at different temperatures through the transitions. The shadow band on each set of plots was placed at the bulk average value with a width equal to the error bars of the data. The regions with extra charge density are indicated by the red arrows.

This “charge rich” and “charge poor” electronic phase separation scenario was theoretically proposed by previous work^37^, but is lacking of direct experimental observations to confirm. Spatially-resolved spectroscopic results, obtained by scanning a small electron beam over the La_1/3_Ca_2/3_MnO_3_ sample, provide further support for the charge segregation scenario. The energy separation *E*_*s*_ of the pre-peak and the main-peak at the oxygen K edge in the electron energy-loss spectra (EELS) has been shown to have a direct correlation to the doping level *x* in doped manganites^38^,^39^. A linear relationship between the *E*_*s*_ and *x* in La_1−*x*_Ca_*x*_MnO_3_ (0 ≤ *x* ≤ 1) (Fig. S5b) is used as a spectroscopic method to quantify the local *x*_*eff*_. Most of the spectra collected at *T* = 300 K as a function of position are the same as the black spectrum shown in Fig. 5a, while a small fraction matches the red one. The sized of the electron probe used for the EELS measurements was ~1.5 nm in diameter, several times the average lattice constants. It follows that the *E*_*s*_ measurements average over a distribution of Mn ions, thus providing an averaged measure of charge density. Using Gaussian curve fitting, *E*_*s*_ were measured to be 6.85 eV and 7.16 eV for the black set and red set of spectra, respectively, giving *x*_*eff*_ to be 0.67 ± 0.04 (the nominal doping of the bulk) and 0.77 ± 0.04, respectively. It is worthwhile to highlight that the *x*_*eff*_ = 0.77 ± 0.04 from a few locations using EELS is quantitatively consistent with the *x*_*eff*_ = 0.75 ± 0.02 obtained using END results. Moreover, the evolution of the regions with extra charge density upon cooling is shown in the line plot of *E*_*s*_ in real-space as a function of temperature (Fig. 5b). The material starts in a homogeneous state at high temperature, consistent with the electronic isotropic phase. Upon cooling, areas with extra charge grow in size and the charge deviation (the value above the shaded band) decreases. The line plot of *E*_*s*_ is back to a homogeneous state at low temperatures, as expected. It should be noted that the value of *E*_*s*_ in the high-temperature homogeneous state (no superstructure) is the same as that in the low-temperature homogeneous state (with LR superstructure), consistent with the expectation that *E*_*s*_ probes the locally-averaged charge state while being insensitive to the structural distortion associated with the orbital ordering. We note that the scanning direction for the displayed data is along the *a* axis; however, there is no qualitative difference observed for other scanning directions. It is very interesting that the results from the END and EELS analyses, which measure distinct local properties, are quantitatively consistent concerning the size, temperature dependence and local charge deviation from nominal doping level, revealing a scenario of electronic phase separation at the nanoscale (see supplemental material for more details) during the ELC phase transitions in La_1/3_Ca_2/3_MnO_3_.

We note that LR antiferromagnetic spin order (see dashed lines in Fig. 3b, which correspond to CE-type chains) was reported in this material to appear at *T* < 150 K^4^,^5^, much lower than both of the ELC phase transitions. Therefore, we ignore the spin effect in the ELC transitions in La_1/3_Ca_2/3_MnO_3_. Based on the proposed model for the incommensurate orbital ordering and the observation of the charge segregation, the C-IC transition could be a result of competing mechanisms arising from the charge-orbital interplay. Specifically, the softening of the orbital excitations, or orbitons, might cause the C-IC transition, while the local charge fluctuation/segregation gives rise to the electronic phase separation and breaks the LR phase into SR. The incommensurate orbital ordering observed here shares common features with some SDW structures. In the latter case, the spin value at each possible location in a SDW is a combination of two spin eigenstates, and SDW systems have been observed to have the C-IC transition, as well. The symmetry breaking and transition in the SDW phase using ELC classification has received considerable attention^18^,^19^,^20^,^21^,^22^,^23^,^24^. Therefore, we expect that the ELC theory can provide more guidance for further exploration of orbitally-ordered structures in correlated materials in the future.

Finally, it is worthwhile to highlight that for La_1/3_Ca_2/3_MnO_3_, the crystalline symmetry decreases from cubic to orthorhombic at 1100 K^40^, indicating that a certain degree of anisotropy between the *a* and *c* directions is already present at high temperature. However, the crystal lattice shows a rapid rise of the anisotropy on cooling below *T*~310 K^4^,^5^ without further symmetry breaking, i.e., retaining the same orthorhombic space group. This rise is clearly associated with the electronic isotropic-nematic transition, driven by electronic correlations, as the change in structural anisotropy is much too large to result from any mechanism driven only by the lattice. A similar situation, involving the growth of nematic order in a crystal with pre-existing broken rotation symmetry is known to occur in YBa_2_Cu_3_O_6+x_^21^,^22^,^41^,^42^,^43^,^44^.

## Methods

The polycrystalline La_1/3_Ca_2/3_MnO_3_ was synthesized using the method in ref. 1. The TEM samples were prepared by routine procedures with mechanical polishing, dimpling and ion milling. Estimated by the EELS results, the sample thickness along the electron beam direction is about 20–30 nm at the areas where the TEM results were obtained. The TEM results, including the dark-field images and the electron diffraction results (SEND maps and END patterns), were obtained within the same single-crystal domain in La_1/3_Ca_2/3_MnO_3_. Results using each of the TEM approaches were obtained with multiple data sets, and representative results are shown.

*In situ* TEM experiments were carried out on a JEOL 3000 F and a JEOL ARM 200 F transmission electron microscope that are equipped with a Gatan liquid nitrogen/helium cooling stage. Electron diffraction patterns were recorded using Fuji imaging plates and obtained by using nanobeam electron diffraction technique with parallel beam. The illumination area is about 200 nm as diameter in the electron diffraction experiments. The peak widths of the SLR’s, shown in Fig. 1a), were first measured from the SLRs and then the values were deconvoluted from the widths measured from fundamental reflections. TEM dark-field images were obtained by tilting the sample to a two-beam condition orientation to strengthen one pair of the SLRs with almost equal intensities around a Bragg peak (202). The image is formed by using those three well-identified reflections, one Bragg peak (202) and two satellite SLRs. Linked to the atomic displacement wave using a periodic model shown in ref. 4, the contrast of the “stripes” in dark-field images can be expressed by the equation: 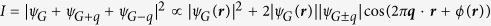, where *ψ*_*G*_(***r***) is the scattering wave function of the Bragg peak (202) and *ψ*_*G*±*q*_ are for the pair of SLRs. Therefore, the contrast of the dark-field images can accurately reflect the periodicity of the superstructure q and the phase discontinuities where the *ϕ*(***r***) term has abrupt changes. For instance, a dislocation can cause a phase shift of π in a propagating atomic displacement wave. More details of the dark-field imaging technique can be found in ref. 9.

## Additional Information

**How to cite this article**: Tao, J. *et al*. Direct observation of electronic-liquid-crystal phase transitions and their microscopic origin in La_1/3_Ca_2/3_MnO_3_. *Sci. Rep*. **6**, 37624; doi: 10.1038/srep37624 (2016).

**Publisher’s note:** Springer Nature remains neutral with regard to jurisdictional claims in published maps and institutional affiliations.

## Supplementary Material

Supplementary Information
