# Supplementary material for: Direct observation of electronic-liquid-crystal phase transitions and their microscopic origin in La1/3Ca2/3MnO3
Source: Sci Rep. 2016 Nov 22;6:37624. doi: 10.1038/srep37624 (PMC5118726; doi:10.1038/srep37624)
Supplement: Supplementary Information [file srep37624-s1.pdf]

Supplementary Information

Direct observation of electronic-liquid-crystal phase transitions and their microscopic origin in $La_{1/3}Ca_{2/3}MnO_3$

J. Tao[1,*], K. Sun[2], W.-G. Yin[1], L. Wu[1], H. Xin[3], J. G Wen[4], W. Luo[5], S. J. Pennycook[6], J. M. Tranquada[1] and Y. Zhu[1]

[1]Condensed Matter Physics & Materials Science Department, Brookhaven National Laboratory, Upton, NY 11973

[2]Department of Physics, University of Michigan, Ann Arbor, MI 48109

[3]Center for Functional Nanomaterials, Brookhaven National Laboratory, Upton, NY 11973

[4]Electron Microscopy Center, Argonne National Laboratory, Argonne, Illinois 60439

[5]Department of Physics & Astronomy, Shanghai JiaoTong University, Shanghai, China

[6]Department of Materials Science and Engineering, National University of Singapore, Singapore, 119077

1. Classification of ELC phases in bulk $La_{1/3}Ca_{2/3}MnO_3$

First of all, we use the high-temperature (above 310 K) crystal lattice of $La_{1/3}Ca_{2/3}MnO_3$ as the fundamental lattice. Although the fundamental lattice is orthorhombic with the space group of *Pnma* ($a \approx c$, $b \sim \sqrt{2}a$) in Ref. [4] and Ref. [5], we still consider the lattice to have C4 rotational symmetry because the lattice constants on the *a* and *c* axes are very close. This is a conventional way of using the classification of ELC phases on other strongly correlated materials such as cuprates YBCO in Ref. [21] and Ref. [22]. Compared to the fundamental lattice, the electronic structure in $La_{1/3}Ca_{2/3}MnO_3$ at low temperatures has LR superstructure that is three times larger than the fundamental lattice along the *a* axis. Thus, the translational symmetry of the electronic structure is *different* than the fundamental lattice, i.e., it breaks the translational symmetry with respect to the fundamental lattice. In the ELC classification, a *broken* translational symmetry is not referring to repeating periodicities as in crystals, but to a different periodicity compared with that of the fundamental lattice. This different periodicity can be either commensurate or incommensurate. However, smectic and nematic orders both refer to electronic phases in bulk materials, namely both have to be LR. Once a LR superstructure melt into separated phases, the electronic structure of the whole material consists of the local phase with the superstructure and other local phase without the superstructure. At this stage, the electronic structure as a whole in the bulk material does not have a different periodicity with respect to the fundamental lattice. Therefore, the translational symmetry of the fundamental lattice is restored in the partially-superstructured electronic phase for the bulk. That is the reason that being LR or SR of the superstructure is critical for identifying translational symmetry breaking in the ELC phases.

On the other hand, identifying rotational symmetry breaking is relatively easier. Since the fundamental lattice is considered to have C4 symmetry, structures along the *a* axis is the same as that along the *c* axis (which is again a reasonable approximation for the study using ELC phases), an electronic phase that has characteristics different along the *a* and *c* axes is identified to have a rotational symmetry breaking from C4 to C2. Based on this definition, as long as the superstructure peaks (no matter being LR or SR) appear on the *a** direction in the reciprocal space as demonstrated in electron diffraction patterns, the rotational symmetry of the electronic phase of the whole material remains broken with respect to the fundamental lattice.

There are four types of electronic phases in the classification of ELC phases [18]. Taking 2D structures having a square lattice (so that the fundamental lattice has a C4 rotational symmetry) as an example, the four types ELC phases are listed as follows: 1) an electronic *crystal* phase has broken rotational symmetry (C2) and translational symmetry breaking along both *x* and *y* directions (*x* and *y* are perpendicular directions and can be orthogonal axes) with respect to the fundamental lattice; 2) an electronic *smectic* phase has broken rotational symmetry (C2) and translational symmetry breaking along one direction with respect to the fundamental lattice; 3) an electronic *nematic* phase has broken rotational symmetry (C2) and no translational symmetry breaking with respect to the fundamental lattice; 4) an electronic *isotropic* phase has no broken

rotational symmetry (C4) and no translational symmetry breaking, i.e., the same structure as the fundamental lattice.

Using the above descriptions, we are able to link the electronic structure in $La_{1/3}Ca_{2/3}MnO_3$ to the ELC phases as a function of temperature. A low temperatures, the LR superstructure has unidirectional stripes with the superstructural periodicity along the *a* axis only. Therefore, it is an electronic smectic phase in the bulk material. At the temperature range that the superstructure only remains in confined nano-regions, the rotational symmetry is C2 and the translational symmetry is the same as the fundamental lattice for the entire electronic structure in the bulk. Thus the electronic phase is a nematic. At the temperatures when the local superstructure disappear completely (with undetectable intensity in the diffraction data), the electronic structure is the same as the fundamental lattice, called an isotropic.

2. Commensurate-incommensurate transition of the superstructure

Electron diffraction results clearly showed a commensurate-incommensurate (C-IC) phase transition through thermal processes in $La_{1-x}Ca_xMnO_3$ ($0.5 \leqslant x \leqslant 0.8$) in Ref. [6] and Ref. [9]. However, the C-IC transition characterized using synchrotron x-ray scattering shows controversy in doped manganites. It clearly stated in Ref. [4] that the periodicity of the superstructure remains three-times as the fundamental lattice upon warming / cooling, i.e., no C-IC transition, using synchrotron x-ray scattering in $La_{0.333}Ca_{0.667}MnO_3$, while the superstructure in similar material system $Bi_{0.33}Sr_{0.67}MnO_3$ was measured to have a very clear C-IC transition upon warming in Ref. [42].

Based on the above results, we argue that the C-IC transition has enough experimental evidences using various techniques. The reason that such a C-IC transition was not observed in Ref. [4] could come from two origins as follows.

1) Upon warming, the superstructure peak becomes broad and diffuse significantly as a function of temperature. This increases the difficulty in accurately measuring the peak position. Moreover, the Borissov's thesis (Chapter 4.2.2, p 57-59 in Ref. [42]) pointed out that the superstructure peak has a change in its shape during the C-IC transition and using symmetric or asymmetric Gaussian-curve fitting can result in different peak-position measurements, which also increases the uncertainty in the characterization. The electron scattering has much stronger scattering form factor than synchrotron x-ray and neutron scattering, and thus could provide much higher intensity of the superstructure peak. This is particularly useful in the characterization when the superstructure peak blurs in intensity and sharpness during the transition. On the other hand, both the electron diffraction results in the manuscript and the synchrotron x-ray results in Ref. [42] were obtained from single crystals (the electron diffraction data were obtained from a volume in a single-crystal domain). This fact may improve the quality of the scattering data for quantitative analysis.

2) Synchrotron x-ray scattering and electron diffraction detect different physical entities in materials. Specifically, synchrotron x-ray scattering are very sensitive to the electron clouds, while electron scatters from both the electron clouds and the nuclei. Therefore, the distinct characterizations of the superstructure using different techniques may partially arise from the nature of the superstructure. Although it is of great interest to

the research community, studying the charge density distribution by comparing the difference coming from the two techniques is beyond the scope of this manuscript.

3. Phase mapping of the dark field images

The intensity of the dark-field images of Fig. 2 can be parametrized as $\propto A\cos(\boldsymbol{q}\cdot\boldsymbol{r}+\varphi(\boldsymbol{r}))$, where A is the amplitude of the superstructure modulation and a constant in the mapping area, and $\boldsymbol{q}$ is the wave vector of the superstructure along the *a*-axis [9]. If the superstructure order retains its coherence over an area, the relative phase $\varphi(\boldsymbol{r})$ should be constant across it. The evolution of the phase function distribution can be found in Fig. S3. However, we note that the sinusoidal assumption used in the phase mapping may not be true. Kinematic diffraction principles can rule out the sinusoidal assumption here if the SLRs at the position ($h \pm nq$, 0, 0) with higher order ($n > 1$) have zero intensity. In order to test the sinusoidal model of the superstructure (see arguments in Ref. [43]), we performed experiments shown in Fig. S4. We conclude that electron diffraction patterns obtained in this work with significant multiple-scattering effect is incapable to identify the sinusoidal model to be true or not. Therefore, with no evidence clearly against the sinusoidal model, we are confident to use it as a reasonable assumption for the phase mapping.

4. Charge segregation / electronic phase separation

The EELS data were collected using an electron probe ~ 1.5 nm in diameter. Namely the electron beam probes the electronic structure and the crystal structure from tens of high-temperature unit cells in the bulk and is unable to reveal the charge periodicity at atomic resolution. The experiment was carried out using a Gatan liquid-He cooling stage/holder with a small amount of instability at low temperatures, i.e., the sample has a drift along certain directions depending on the sample mounting. This drift prevents us to have atomic resolution in the EELS results but does not affect the data acquisition / analysis at nanoscale level. We performed the EELS line-scan at various scanning directions, either parallel or perpendicular to the stripe direction. We found that there is no measurable change between data along the two scanning directions.

The charge segregation is limited by the Coulomb energy cost. The Coulomb energy cost was estimated to be less than 10 meV by considering all the following facts. The dielectric constant of the system is reported to be higher than 40 during the transition (see Ref. [44]). We assumed a spherical shape for the nano-regions with the superstructure order with measured size ~ 5 nm and volume fraction roughly ~ 3 % at 300 K. The charge deviation of ~ 0.1 holes per unit cell was deduced by EELS and END at room temperature. This estimation of the Coulomb energy cost is on the same order as the thermal energy, suggesting that it is energetically feasible to sustain the observed nanoscale electronic phase separation.

To maintain the charge neutral of the bulk $La_{1/3}Ca_{2/3}MnO_3$, the disordered regions have to have less charge density (extra electron density) than the nominal doping. However, it would be very difficult for us to probe that. Firstly, the disordered regions

provide no SLRs in the diffraction patterns. Secondly, by considering the volume fraction of the ordered and disordered regions, the extra electron density in the disordered regions is smaller than the accuracy of our EELS technique. For example, the ordered regions has volume fraction of roughly 3 % with a ~ 0.1 extra holes per Mn site at T = 300 K, then a 0.003 extra electrons per Mn site could be inferred in the disordered region. The situation remains the same at lower temperatures because the ordered areas have increased volume fraction but decreased charge density deviation.

5. Ginzburg–Landau free energy and simulations of the dislocation pairs

Here, we consider a discrete model defined on a 2D square lattice. It must be emphasized that although this is a 2D model instead of 3D; within the mean-field approximation, 2D and 3D models produce the same qualitative conclusions.

Because the system is an insulator, hopping of the electrons/holes can be ignored. Within the mean-field approximation, the Ginzburg–Landau free energy only relies on the charge density on each site

$$F = \sum_{i,j} [a\, \rho_{i,j}\rho_{i,j} + \left(b_x\rho_{i+1,j}\rho_{i,j} + b_y\rho_{i,j+1}\rho_{i,j}\right) + \left(c_1\rho_{i+1,j+1}\rho_{i,j} + c_2\rho_{i+1,j-1}\rho_{i,j}\right) + \left(d_x\rho_{i+2,j}\rho_{i,j} + d_y\rho_{i,j+2}\rho_{i,j}\right) + g\, {\rho_{i,j}}^3 + u\, {\rho_{i,j}}^4 - \mu\, \rho_{i,j}]$$

where i and j are two integers labeling the 2D coordinate for each lattice site and $\rho_{i,j}$ is the charge density on the site (i,j). The first term in the Ginzburg–Landau free energy is the on-site repulsion between particles occupying the same site. The second, third and fourth terms describe the nearest-, next-nearest- and next-next-nearest neighbor repulsions respectively, while longer range interactions are ignored in this model. The quartic term $u\, {\rho_{i,j}}^4$ is necessary for stability reasons as will be discussed below. A cubic term $g\, {\rho_{i,j}}^3$ is also added, since it is allowed by symmetry. Here, we choose to work in the grand canonical ensemble, which is more convenient for minimizing the Ginzburg–Landau free energy, and thus the last term is added, where μ is the chemical potential.

For a square lattice with 4-fold rotational symmetry, the coefficients must satisfy the following symmetry constraints: $b_x = b_y$, $c_1 = c_2$ and $d_x = d_y$. However, in electronic liquid crystal phases, nematic ordering introduces anisotropy and thus removes some of these constraints. For example, a main-axis nematic order, which breaks the symmetry between the x and y axes, only requires $c_1 = c_2$, while $b_x \neq b_y$ and $d_x \neq d_y$ in general. Here, the difference between b_x and b_y (or d_x and d_y) measures the amplitude of the nematic ordering. Similarly, for a diagonal nematic order, which breaks the symmetry between the $x + y$ and $x - y$ directions, the following two relations must hold $b_x = b_y$ and $d_x = d_y$, but c_1 and c_2 can take different values and the difference between them measures the strength of the diagonal nematic ordering. For our system, because only the main-axis nematic ordering arises, we require $c_1 = c_2 = c$ and $b_x \neq b_y$.

By minimizing the Ginzburg–Landau free energy numerically, the density configuration can be obtained. But before the numerical results are presented, we first discuss the qualitative properties of this model and demonstrate that the free energy

describes a transition from a homogeneous phase into a stripe phase with a unidirectional charge modulation. By rewriting the free energy in the momentum space, we find that

$$F = \sum_{\vec{q}} \alpha_{\vec{q}} \rho_{\vec{q}} \rho_{-\vec{q}} - \mu N + O(\rho_{\vec{k}})^3$$

where $\vec{q} = (q_x, q_y)$ is the 2D momentum vector and the coefficient $\alpha_{\vec{q}}$ is

$$\alpha_{\vec{q}} = a + b_x \cos q_x + b_y \cos q_y + c[\cos(q_x + q_y) + \cos(q_x - q_y)] + d_x \cos 2q_x + d_y \cos 2q_y$$

and $\rho_{\vec{q}}$ is the Fourier transformation of $\rho_{i,j}$. The $\vec{q} = 0$ component ($\rho_{\vec{q}=0}$) is the average density of the system and each $\vec{q} \neq 0$ component ($\rho_{\vec{q}\neq 0}$) describes a density wave with wavevector $\vec{q}$. If $\alpha_{\vec{q}} > 0$ for all $\vec{q} \neq 0$, to minimize the free energy, we must have $\rho_{\vec{q}} = 0$ for all nonzero $\vec{q}$. As a result, the system is in the homogeneous phase with charge distributed uniformly on each site. If a certain $\alpha_{\vec{q}}$ takes a negative value, the homogeneous state will no longer minimize the free energy and thus becomes unstable, resulting in an inhomogeneous state. Very typically, the charge modulation in the inhomogeneous phase is dominated by the most unstable mode, whose wavevector $\vec{Q}$ makes $\alpha_{\vec{q}}$ reach the most negative value. In other words, the inhomogeneous phase shall contain a unidirectional charge modulation, whose wavevector is close to $\vec{Q}$.

In our study, we choose $a = 3.42$, $b_x = -1.28$, $b_y = -1.12$, $c_1 = c_2 = 0.8$, $d_x = d_y = 0.2$. The chemical potential is $\mu = 1.6$ and we set $g = -2.4$ and $u = 1$. By minimizing the free energy on a 21×30 square lattice with periodic boundary conditions, a unidirectional charge modulation with $\vec{Q} = \left(\frac{2\pi}{3}, 0\right)$ is observed.

For a perfect crystal, the nematic order parameter is expected to be uniform. In our model, as has been shown above, the nematic order parameter is measured by the difference between b_x and b_y, which shall be a constant in a clean system. However, in a real material, due to lattice defects and other material inhomogeneity, local domains may arise, in which the nematic order parameter takes different values and even the opposite sign. Here, we introduce such a domain (of size 4×12) by flipping the values of b_x and b_y inside the domain. By numerically minimizing the free energy, we find that the domain created a pair of dislocations for the charge modulation. Between these two dislocations, a nanometer-size patch arises, in which the charge modulation vanishes (melts), in analogy to the experimental observation.

Because the amplitude of the charge modulation inside and outside of the domain is different, the charge density inside and outside the domain must also be different. This effect comes from nonlinear terms in our theory. For example, if we rewrite the cubic term in the momentum space, the following term will arise $g\, \rho_{\vec{q}=0} \rho_{-\vec{Q}} \rho_{\vec{Q}}$, where $\rho_{\vec{q}=0}$ is the average density and $\rho_{\pm\vec{Q}}$ is the order parameter of the charge modulation. If $|\rho_{\pm\vec{Q}}| \neq 0$, this term will renormalize the chemical potential ($\mu \to \mu - g|\rho_{\vec{Q}}|^2$). This effect implies that the amplitude of the charge modulation has a direct impact on the chemical potential. Therefore, the charge density inside the domain, where the charge modulation is weak, differs from the rest of the system, where the charge modulation is strong. If we perform

the same analysis for the quartic term, we will find that there the amplitude of the charge modulation renormalizes the compressibility, which also results in different charge densities inside and outside the domain.

To verify this conclusion, we calculated the local average density for the model above by averaging the charge density inside each 3×3 block. As shown in Fig. 4(b), indeed the charge density inside the domain is about 10% lower than outside.

6. Supplemental figures

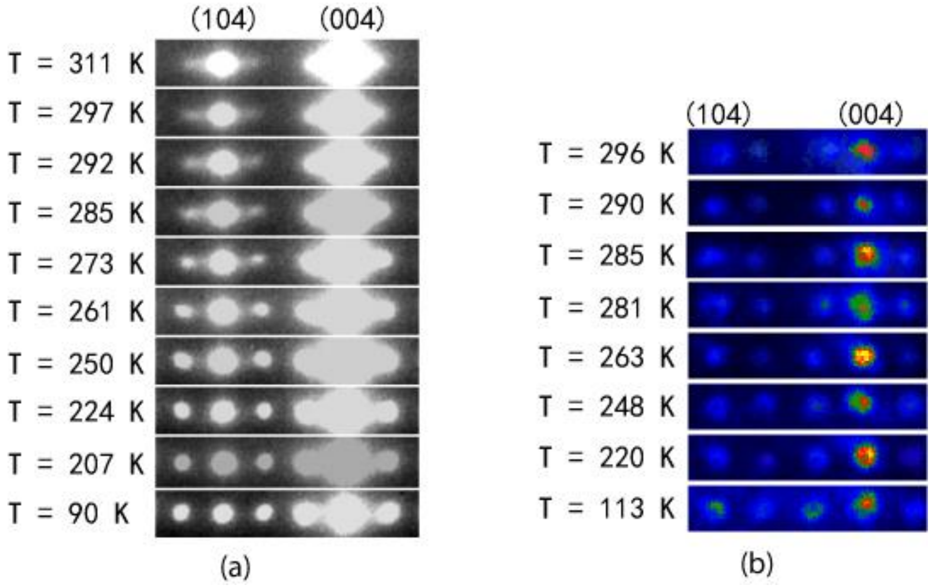

Figure S1. (a) The evolution of the SLRs from ED patterns at the [010] zone axis obtained from a single-crystal domain upon warming. (b) The evolution of the SLRs from END patterns at the [010] zone axis obtained from a single-crystal domain upon warming. Both evidently show a low-temperature wave number ~ 1/3 of a* in the reciprocal space and the position of the SLRs moves through the transition, i. e., a commensurate-incommensurate transition of the superstructure.

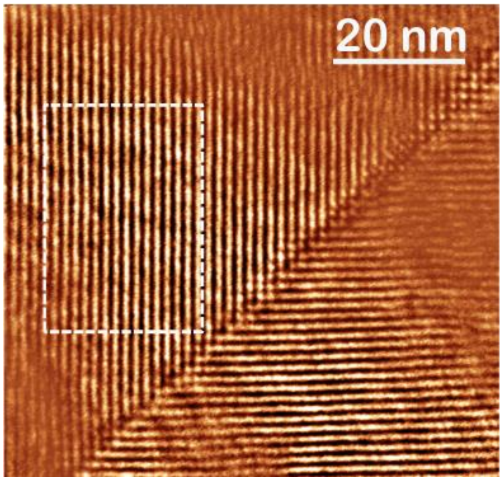

Figure S2. A dark-field TEM recorded at T = 98 K in $La_{1/3}Ca_{2/3}MnO_3$ with crystalline domain boundary. The dash box shows the area where the TEM images in Fig. 2 were obtained.

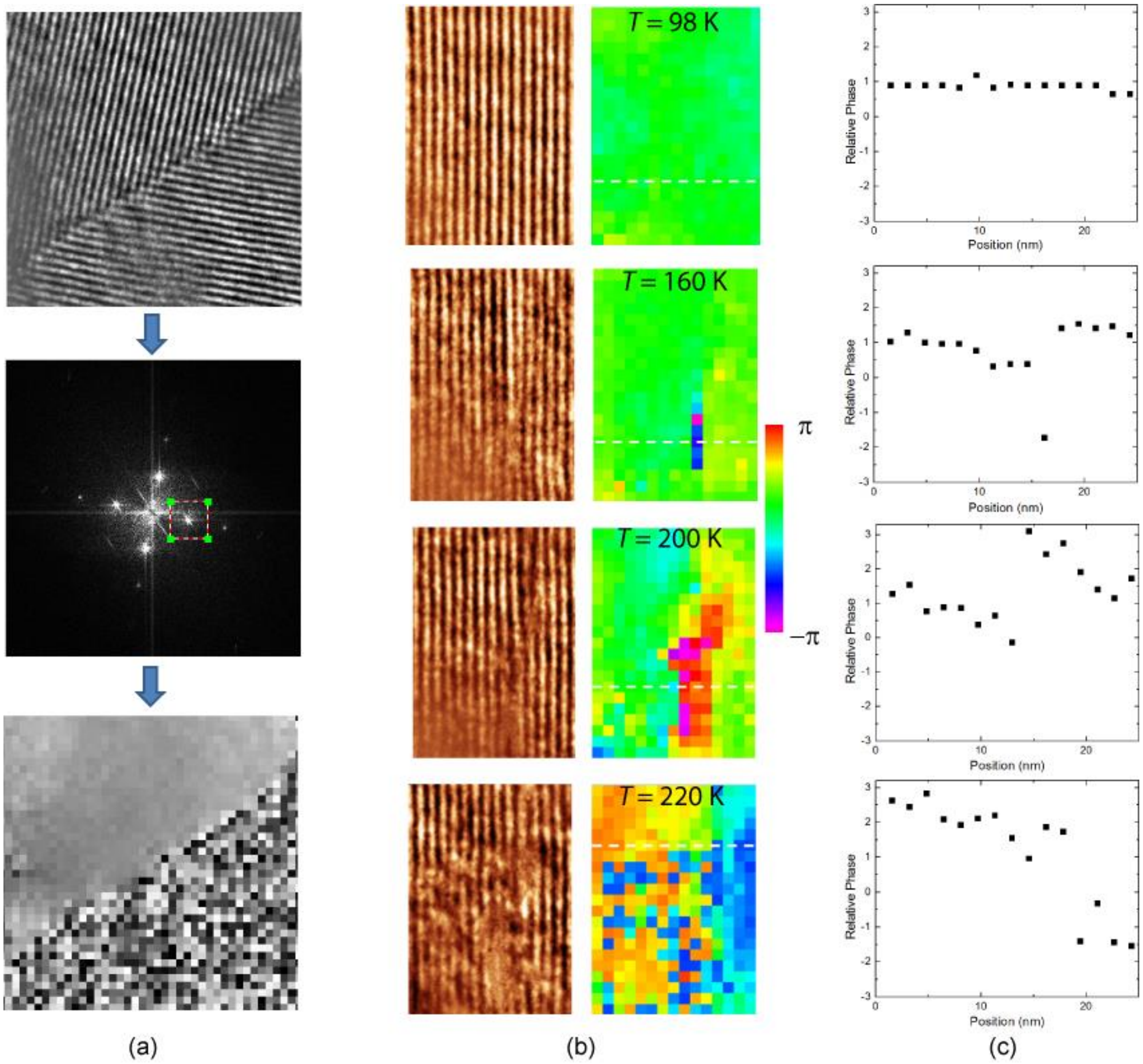

Figure S3. (a) Taking a dark field image (top) as an example, the phase mapping procedure is demonstrated by performing fast-Fourier transform (middle), selecting side band as the dash box in the fast-Fourier transform, reconstructing phase map (bottom) using the Gatan DigitalMicrograph software. The image has two crystal domains with superstructures orthogonal to each other. By selecting one side band in the dash box, the mapped phase is uniform (on grey scale) in the domain with corresponding superstructure and the other domain has randomly distributed phase. (b) The phase maps using the dark field images in Fig 2 in the manuscript, with a color scale shown on the right. The amplitude profile of the phase function on the dash line at each temperature is shown on (c). Clearly the discrete dislocation pairs are the singularities in a uniform phase map, but the proliferation of the dislocations changes the coherency of the superstructure.

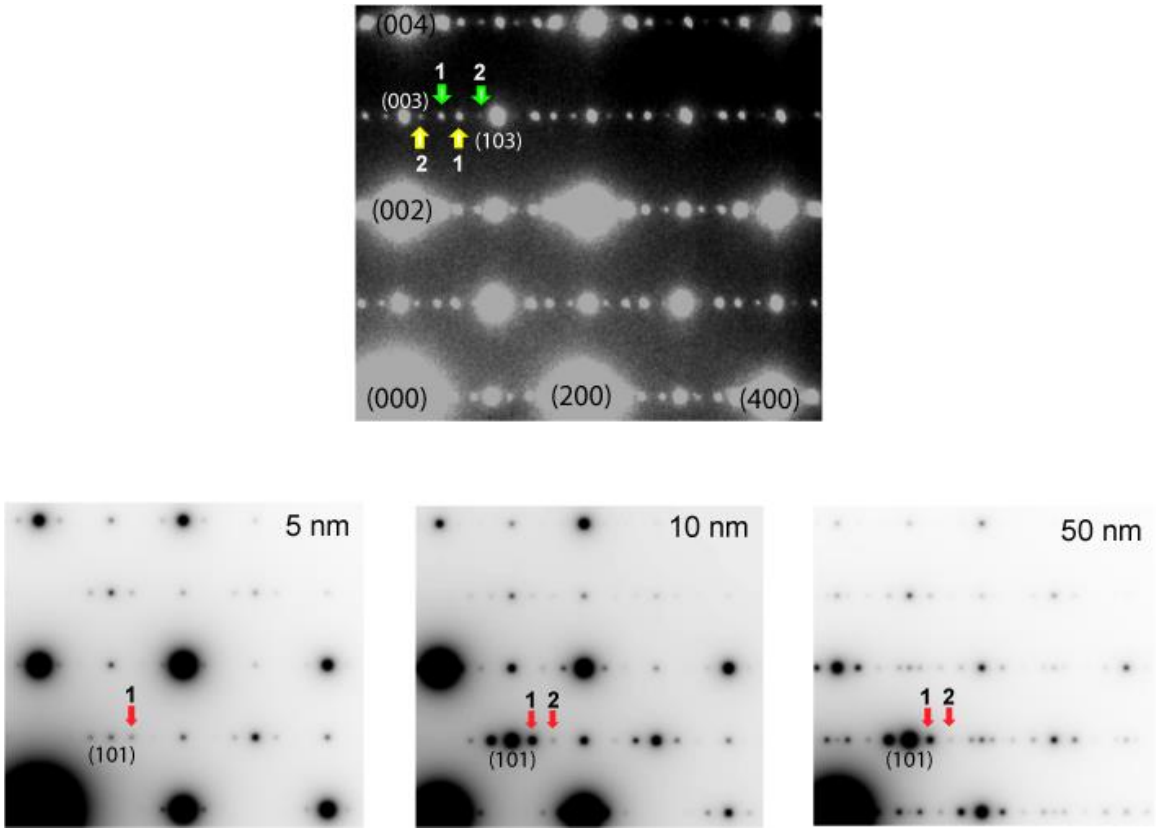

Figure S4. Top: An experimental electron diffraction pattern obtained from $La_{0.42}Ca_{0.58}MnO_3$ at temperature of 100 K at the [010] zone. Superlattice reflections (SLRs) corresponding to the superstructure can be seen around the fundamental reflections. The green arrows highlight the first-order SLR (n = 1) and the second-order SLR (n = 2) around the fundamental reflection (003), while the yellow arrows highlight the first-order SLR and the second-order SLR around the fundamental reflection (103). Bottom: Simulations using the Bloch wave method for electron diffraction patterns obtained in $La_{1-x}Ca_{x}$MnO3 with an incommensurate superstructure at different sample thicknesses.

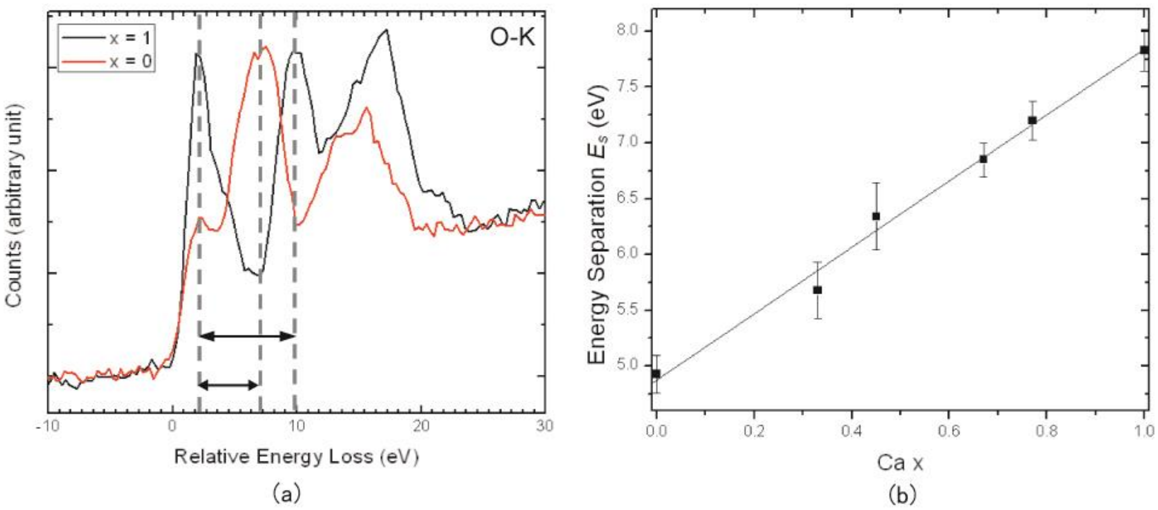

Figure S5. (a) Two examples of the EELS at the oxygen K edge obtained from room-temperature $LaMnO_3$ and $CaMnO_3$, clearly showing a different energy separation between the pre-peak and the main peak in the spectra. (b) E_s as the two peak energy separation measured from the EELS at the oxygen K edge as a function of doping level x in $La_{1-x}Ca_xMnO_3$ ($0 \leq x \leq 1$) samples, with good linear fit for the plot. Each measurement was averaged out over the sample at the particular doping level at room temperature.
